# SWORD2: hierarchical analysis of protein 3D structures

**DOI:** 10.1093/nar/gkac370

**Published:** 2022-05-17

**Authors:** Gabriel Cretin, Tatiana Galochkina, Yann Vander Meersche, Alexandre G de Brevern, Guillaume Postic, Jean-Christophe Gelly

**Affiliations:** Université Paris Cité and Université des Antilles and Université de la Réunion, INSERM, BIGR, F-75015 Paris, France; Laboratoire d’Excellence GR-Ex, 75015 Paris, France; Université Paris Cité and Université des Antilles and Université de la Réunion, INSERM, BIGR, F-75015 Paris, France; Laboratoire d’Excellence GR-Ex, 75015 Paris, France; Université Paris Cité and Université des Antilles and Université de la Réunion, INSERM, BIGR, F-75015 Paris, France; Laboratoire d’Excellence GR-Ex, 75015 Paris, France; Université Paris Cité and Université des Antilles and Université de la Réunion, INSERM, BIGR, F-75015 Paris, France; Laboratoire d’Excellence GR-Ex, 75015 Paris, France; Université Paris-Saclay, Univ Evry, IBISC, 91020 Evry-Courcouronnes, France; Université Paris Cité and Université des Antilles and Université de la Réunion, INSERM, BIGR, F-75015 Paris, France; Laboratoire d’Excellence GR-Ex, 75015 Paris, France

## Abstract

Understanding the functions and origins of proteins requires splitting these macromolecules into fragments that could be independent in terms of folding, activity, or evolution. For that purpose, structural domains are the typical level of analysis, but shorter segments, such as subdomains and supersecondary structures, are insightful as well. Here, we propose SWORD2, a web server for exploring how an input protein structure may be decomposed into ‘Protein Units’ that can be hierarchically assembled to delimit structural domains. For each partitioning solution, the relevance of the identified substructures is estimated through different measures. This multilevel analysis is achieved by integrating our previous work on domain delineation, ‘protein peeling’ and model quality assessment. We hope that SWORD2 will be useful to biologists searching for key regions in their proteins of interest and to bioinformaticians building datasets of protein structures. The web server is freely available online: https://www.dsimb.inserm.fr/SWORD2.

## Graphical Abstract

**Graphical Abstract:**
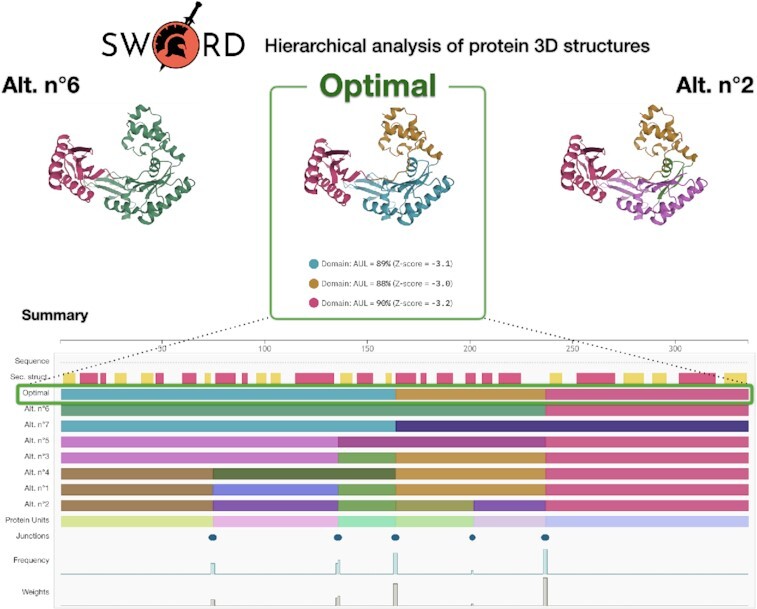
SWORD2 webserver proposes an interactive visualisation and analysis of multiple alternative hierarchical organizations of protein structures in terms of domains, protein units and secondary structures, assessed by different metrics.

## INTRODUCTION

The first step of protein structure analysis usually consists in breaking down the complex protein structure into simpler yet structurally and functionally relevant units called structural domains that can be further characterized individually. Elementary units of protein structures have already been identified at different levels: starting from several residues in case of secondary structures, to several tens of residues in domains, as well as elements of intermediate size called super secondary structures or Protein Units (PUs) (1,2). The identification of secondary structure elements is quite straightforward and follows a well established set of geometrical and physical rules (3,4). However, the determination of higher level structural units remains challenging because of its inherent ambiguity (5).

The annotation of protein structures at different levels is crucial in essentially all fields trying to analyze different aspects of proteins, such as their function, folding, flexibility, evolution, interaction, as well as fields tackling computational prediction and design. For that reason, several methods for the detection of protein domains were developed in the last three decades (6). Some of them are based on libraries of structural templates such as CATHEDRAL (7), or use clustering of substructures (DIAL (8)). Others are template-independant and based on structural characteristics of domains: DomainParser (9), PDP (10), DDOMAIN (11), DHcL (12); or on predicted domains from sequence: SnapDRAGON (13), RosettaDOM (14), OPUS-Dom (15), DNN-Dom (16) and FUpred (17). Such a wide variety of methods that strive to determine protein domains illustrate the fact that there is no authoritative definition of protein domains. Each method was developed to tackle a problem according to one of multiple definitions of a domain. In fact, such ambiguity has been characterized by Postic *et al.* (5) and shows that different protein decompositions can lead to equally valid domain annotations. This is why the SWORD (5) algorithm was developed in order to propose multiple alternative domain assignments for a given protein structure. SWORD aims at handling ambiguous cases of protein structure partitioning. The decomposition of the protein structure into domains is achieved through the hierarchical clustering of Protein Units (1,2), which are evolutionarily preserved (18) structural descriptors of intermediate size, between secondary structures and domains. These compact fragments are determined by Protein Peeling algorithm (2) following two main rules: they must have high intra-PU contacts and low inter-PU contacts. PUs are more elementary structural descriptors than domains, but are large enough to be structurally and evolutionarily relevant, making them perfect candidates to form higher level structural domains. SWORD algorithm reaches comparable or superior agreement with annotations from data banks compared to other methods and is the only method providing protein decomposition at different hierarchical levels.

We present SWORD2, a web-based server that allows users to explore a hierarchical decomposition of protein structures at different levels to help better understand and analyze the organization of protein structures. Combining both Protein Peeling (PP) (2) and SWORD (5), we offer a three-level view of protein architecture: secondary structures, PUs and Domains. SWORD generates multiple domain delineations based on a top-down/bottom-up algorithm using PUs. The web server interface allows users to dynamically select the different domain partitionings, their corresponding PUs and visualize them in both the sequence and 3D Viewer. Contact probability maps are provided for every PU and domain generated by both PP and SWORD. A qualitative assessment (5) is provided for each domain assignment with a visual indicator ranging from 1 to 5 (the higher, the better). Another measure called the A-index (Ambiguity index) (5) assesses the overall ambiguity of the protein in terms of possible domain assignments. A probabilistic assessment of each PU and domain individually is also provided to estimate their ability to fold autonomously, based on our recent information-gain approach (19).

## METHODS

### Web server implementation

The SWORD2 web server frontend is built statically using the Bootstrap framework, HTML5, CSS3 and Javascript. The PDBe implementation of Mol* (https://github.com/molstar/pdbe-molstar) was used for the 3D protein viewer. The Features Viewer was developed by the RCSB PDB group, Saguaro 1D Feature Viewer project (https://github.com/rcsb/rcsb-saguaro) (20). Plotly JS was used for the histogram representation (https://plotly.com/javascript/). Fetching, parsing and analysis of protein structures was done with Prody (21–23). The backend is managed by Python3 and user submissions are handled by Python CGI. The approximate job runtime depends on the protein length and its complexity and is estimated at the time of the submission. A typical job for a 150 residues protein usually lasts <1 min.

### SWORD2 algorithm

SWORD2 web server takes a protein structure coordinates file in PDB (24) format as input. Then, SWORD2 proceeds to the hierarchical analysis following the steps described below.

#### Secondary structure assignment

The first hierarchical level of analysis proposed by SWORD2 is the secondary structures. They are assigned using DSSP (4,25). The 8 conformational states defined by DSSP are reduced to three states according to: H (Helix, red) = {H, G, I}, E (Sheet, yellow) = {E, B} and C (Coil, empty spaces between H and E segments) = {S, T, C}.

#### Protein peeling

The second step of analysis proposed by SWORD2 is the Protein Units (PUs). Protein Peeling (1) identifies PUs using an iterative hierarchical clustering of the contact probability map according to two main rules: PUs should be characterized by a high number of intra-PU contacts and a low number of inter-PUs contacts. Contact probabilities between residues are computed from the distances between C_α_ atoms using a logistic function in order to avoid hard threshold cut-off for definition of the inter-residue contacts.

#### SWORD

The third analysis is determined by SWORD, which uses the PUs identified by Protein Peeling to reconstruct domains by gradually merging them until finding the optimal domain delineation based on separation (*S*) and compactness (*C*). High value of *S*_*i*,*j*_ indicates a high number of contacts between PUs *i* and *j*, meaning that these PUs are good candidates to be merged. The compactness criterion *C*_*i*,*j*_ measures the contact density of the protein domain resulting from merge of PUs *i* and *j*. A high value indicates that merging PUs *i* and *j* is a favorable event. Therefore, SWORD returns all the domain partitions falling in the acceptance range of separation and compactness values. Acceptable delineations at a given level of partionning (e.g. two domains, three domains) are sorted according to the compactness criterion of the domains *C*. Finally, the optimal partitioning is selected as the one which (i) has the largest number of domains, (ii) falls in the acceptance range (based on *S* and *C*) and (iii) has the highest compactness compared to the other delineations at this level.

#### Assessment of partitions quality

The quality measure of the obtained partitions is calculated using a step function of distances which divides the space of solutions into acceptable and non-acceptable decompositions (5). The discrete value ranges from 1 to 5, so that a decomposition with a quality of 5 defines domains that are highly compact and separated.

#### Assessment of domains native-like character

To estimate the likelihood that a delimited domain is an independent protein fragment (in terms of folding, stability or evolution), SWORD2 uses our recent *Total Information Gain* (TIG) scoring method (19), which allows to predict the native-like character of a 3D structure. As a model quality assessment program, this score is designed to behave like the Gibbs free energy and is often referred to as ‘pseudo-energy’. Based on pairwise distances, the value of the TIG tends to increase with increasing macromolecule size. To overcome this bias, a *Z*-score of the TIG is computed, following the atom shuffling method (26): for each domain, 2000 random sequence decoys are generated, the pseudo-energies of which follow a non-normal distribution of parameters *μ* and *σ*. The *Z*-score is calculated as (*E* − *μ*)/*σ* and, therefore, expresses the distance (in standard deviations *σ*) between the pseudo-energy of the domain *E* and those of the random decoys. To provide users a direct interpretation of the *Z*-score value, SWORD2 outputs the probability estimated by Chebyshev's inequality that we call *Autonomous Unit Likelihood* (AUL). Thus, for a substructure with a *Z*-score of −2.0, the probability of not observing the same pseudo-energy for a randomly delineated domain reaches 75%. This probability of being native reaches 94% for a domain with a *Z*-score of −4.0. The higher the probability, the more likely it is that the region under consideration is capable of autonomous folding.

#### Ambiguity index

A-index is a measure of structural ambiguity that we devised in SWORD’s seminal article (5). It is a quantitative characteristic related to the number of valid domain decompositions of the protein structure. It therefore accounts for the complexity of the protein structure organization and the difficulty of clearly and unambiguously identifying structural regions of the protein. It represents a balance between the number of identified partitions and the quality of their domains. A protein has an A-index of *A* if *A* of its *N_d_* domain partitions have a quality of at least *A*, and the other (*N_d_* – *A*) domains each have a quality lower than *A*. Therefore, a low *A*-index is neither good nor bad, as a protein with few high-quality domains and a protein with many low-quality domains will both have a low A-index.

### Web server description

#### Submission

The web server is completely free to use for any user without login. Users can either provide a PDB code, or upload a structure file (PDB format, accepted extensions are .pdb and .ent) and specify a protein chain. The user can provide an email address to receive the results when computation is completed.

If the uploaded file does not correspond to a valid PDB structure or the provided PDB code is incorrect, the job is canceled with an explicit error message.

SWORD2 and its dependencies require a protein chain name to be provided, otherwise chain *A* is assigned by default. Once the file is fully parsed, non-standard residues, heteroatoms and residues having insertion codes are removed and the residues are renumbered starting from 1. As a consequence, users should note that any gap in the original PDB file would not appear in the results page.

Finally, a user can also submit an ID from the AlphaFold Protein Structure Database (27,28) (https://alphafold.ebi.ac.uk/) by using the field ‘AlphaFold Uniprot Accession Id’.

#### Output summary

The summary allows users to have a quick and interactive overview of the SWORD2 results. It shows all the identified domain decompositions of the query protein. First the optimal one, followed by decompositions sorted by the least (top) to highest number of domains (bottom). We also highlight the inter-PUs junctions (‘Junctions’, ‘Counts’, ‘Frequency’ and ‘Weights’), corresponding to the extremities of the different PUs used by SWORD to build the domains. ‘Counts’ is the number of times a junction is found among all SWORD2 alternative partitions. ‘Frequency’ represents the junction count, standardized by the total number of domain delineations produced. ‘Weights’ represents the frequency weighted by the delineation quality and standardized by the total number of identified domain delineations. Domain delineations and Protein Units delineations are clickable. A click on a domain or a PU (colored rectangle) triggers a notification for the user and makes 3D viewer focus and highlight the selected domain or PU delineation, while simultaneously coloring the corresponding sequence fragment (Domain or PU) on top of the viewer and updating the contact probability map in the ‘Contact probability maps’ tab, with either all the PUs used to build the domain or the single PU selected.

Two different color sets are used consistently in the results page. Domains are assigned a set of dark tone colors while PUs are represented by a set of lighter tone colors to better distinguish both types.

These colors are used in the Summary, the 3D viewer (Sequence + 3D Protein view), the partitions section (Domains & Protein Units), the contact probability maps, and the histogram. Thus, a domain colored in orange will always be referenced with the same color on the results page. Same rule applies for Protein Units.

#### Details of domain and PUs

For each identified domain decomposition, the aforementioned quality measure is represented by a color bar gauge ranging from one to five (five is best). Users can visualize a certain SWORD partition by clicking on its dedicated display button which will color the protein domains accordingly in the 3D Viewer as well as in the sequence viewer.

For each identified domain, the ranges of residue numbers of the PUs composing them, together with their respective aforementioned AUL score and *Z*-score, are provided. Also, like in the Summary, clicking on an individual domain or PU (color badge) will focus and color it in the 3D viewer and also update the contact probability map tab with the corresponding figure (see ‘Contact probability map’ section). The PU(s) that compose a domain are unveiled when clicking on it as well as their corresponding AUL score and *Z*-score.

#### Peeling levels

The ‘Protein Units’ tab shows the exhaustive set of PUs determined by the Peeling algorithm at different levels. A low Peeling level reveals few and rather long PUs, whereas higher Peeling levels present more and shorter PUs. Colored badges represent the residue range of PUs that were used by SWORD for the hierarchical construction of the domains of the different alternative partitionings, while a black color badge means it was unused.

For each Peeling level, an entropy-derived measure allows the assessment of PUs compactness: the Compaction Index (CI) (1). This index focuses on the non-local contacts in the PUs. For each PU, SWORD2 also provides its likelihood of functioning as an independent substructure, through the calculation of the TIG score.

#### Junctions

Junctions correspond to the extremities of the different Protein Units used by SWORD to build the domains. The junctions correspond to zones of probable cuts in the structure, whether these cuttings are of structural, evolutionary or functional origin. The ‘Junctions’ tab provides detailed information on their respective positions in the sequence, the number of times they are found among all SWORD partitionings, their frequencies and weighted frequencies (see ‘Output summary’ section). The statistics of junctions extracted from all SWORD partitionings can bring interesting insights over the quality of delineations, such as the robustness of domains or on the contrary, highlight the ambiguity underlying some portions of the protein which can not be unequivocally defined.

#### Interactive visualisation

The 3D protein viewer is built using the PDBe's implementation of Mol* (29) with a large panel of features. Users can freely manipulate the protein structure, as well as download a high definition image of a customized protein representation. Users can select residues either in the sequence on top or directly on the structure. When a user submits an AlphaFold Uniprot Accession Id, the 3D viewer will display the corresponding CIF structure file from the AlphaFold Protein Structure Database which includes the Confidence Score.

#### Contact probability map

The contact probability maps of PUs, domains and SWORD partitions are provided under the ‘Contact probability maps’ tab. All figures can be accessed inside the downloadable archive of the results. The PUs and domains are traced on the figure using the same color as the one they refer to in the whole results page for consistency (see ‘Protein Peeling’ section).

#### Protein domains frequency

The count of domains in all the identified alternative SWORD partitionings is represented as an histogram under the ‘Frequency of Protein Domains identified by SWORD’ section. Bars are ordered by descending frequency from left to right allowing users to quickly identify the most frequently occuring domains, which are potentially the most robust. The Plotly interactive plot allows users to select a particular domain (color bar). Users can use the tools of the plot to select particular columns, zoom in a particular region, reset the view, as well as download the plot as a .png file.

## RESULTS AND DISCUSSION

Multiple domain decompositions and different metrics provided by SWORD2 are particularly interesting for the analysis of proteins with complex structure-function relationships often reflected by multiple domain annotations in different databases. Here we present two examples of such proteins and biological insights provided by SWORD2.

### Example 1: DNA polymerase IV (PDB: 1jx4A)

**Figure 1.**
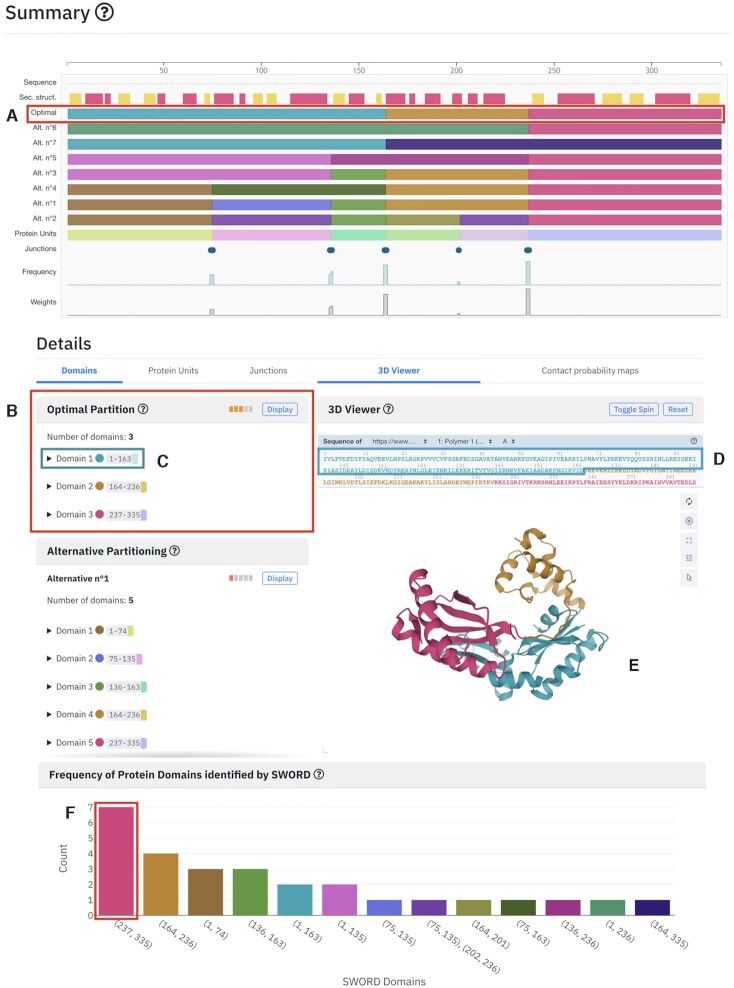
SWORD2 results page example for DNA Polymerase (PDB: 1jx4). (**A**) In the Summary overview user can see and select a SWORD partition or PU. The optimal partition has three domains colored distinctly. (**B**) On the left panel of the Details section, the corresponding optimal partition presents the different domains of the partition with their respective AUL score and Z-score. Each Domain line is collapsible to show in detail the PUs used to build the Domain with their respective AUL and *Z*-score as well. (**C**) Each domain and PU color badge is clickable and will highlight and focus the corresponding sequence and structure segment in the 3D Viewer on the right panel. (**D**) The sequence of the Domain 1 of the optimal partition is colored the same as the domain color badge. (**E**) The Domain is focused in the 3D viewer and colored consistently. (**F**) Focus on the most consistent domain on the panel displaying the domains consistency.

For DNA polymerase IV (PDB: 1jx4) there exists a number of different domain annotations in manual and semi-manual data banks. SWORD2 successfully identifies all of them in terms of Jones criterions (30): the two domains from SCOP/SCOPe/SCOP2 (31–35) (Figure 1. Domain alternative no. 6 (Alt. n°6)), the three domains from CATH 3.4 (36) (Figure 1. Optimal Domains) and four domains annotated from CATH since version 4.0 and ECOD (37,38) (Figure 1. Domains alternative no. 4 (Alt. n°4)). These organizations correspond to different levels of organization and/or biological concepts.

Moreover, quality measures assigned by SWORD2 also correlate perfectly with the information contained in the explored databases. Indeed, all the annotations from CATH, SCOP and ECOD identified the region 237–335 as a domain and the consistency histogram reported by SWORD2 (Figure 1F) indicates high confidence of this domain assignment.

### Example 2: Trp repressor

**Figure 2.**
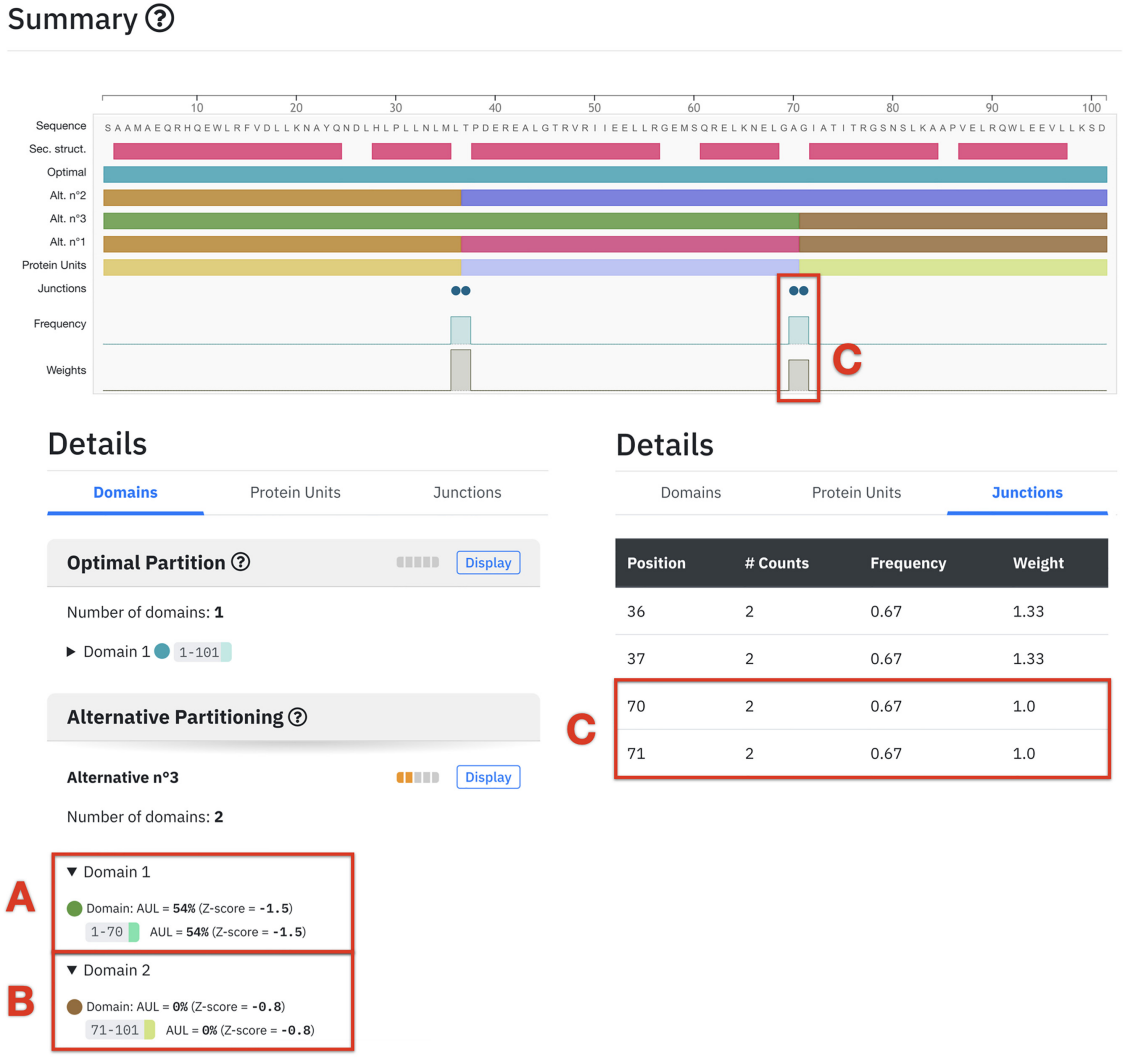
SWORD2 results page example for Trp repressor (PDB: 1jhgA).

SWORD2 also provides important insights for the analysis of the Trp repressor (PDB: 1jhgA), in particular thanks to the AUL assessments and junction evaluation illustrated in figure 2. Among all domains found in alternative partitions, the one with the highest AUL (near 55%; Figure 2A) is obtained for the 1–70 domain. Importantly, this is the only domain whose ability to fold independently has been demonstrated experimentally in contrast to the 71–101 domain, which is unable to fold independently and displays the lowest AUL score: 0% (*Z*-score of –0.8; Figure 2B). Finally, the highly significant value of the main junction near the position seventy (Figure 2C) corresponds to the only chymotrypsin cleavage site experimentally observed between the fifty potential cleavage sites in the core protein (39).

## SUMMARY

SWORD2 is a web server, which brings together several methods that we previously developed to annotate protein structures at different architectural levels: secondary structures, intermediate-sized independent units called Protein Units and domains.

SWORD2 proposes multiple and valuable possible delineations of domains, enables a hierarchical visualization of protein structures, and provides insights into difficult and ambiguous cases. The additional statistics on junctions delimiting Protein Units and domains deliver new meaningful protein annotations. Finally, an estimation of the ability of each defined Protein Unit and domain to fold autonomously is also proposed.

## DATA AVAILABILITY

The web server is freely available online without login requirement: https://www.dsimb.inserm.fr/SWORD2.

## Notes

Present address: Jean-Christophe Gelly, INSERM, UMRS_1134, DSIMB, 6, rue Alexandre Cabanel, 75739 Paris Cedex 15, France.
